# Association of hospital-initiated bone densitometry with hospitalization for fragility fracture at Lille University Hospital among adults with chronic obstructive pulmonary disease

**DOI:** 10.1007/s11657-025-01534-3

**Published:** 2025-04-09

**Authors:** Eléonore Heddebaut, Pierre Balayé, Emeline Cailliau, Olivier Le Rouzic, Cécile Philippoteaux, Julien Paccou

**Affiliations:** 1 Department of Rheumatology, Univ. Lille, CHU Lille, 59000 Lille, France; 2 Public Health Department, Univ. Lille, CHU Lille, ULR 2694 - METRICS, CERIM, 59000 Lille, France; 3 Biostatistics Department, CHU Lille, 59000 Lille, France; 4 Institut Pasteur de Lille, Univ. Lille, CHU Lille, CNRS, Inserm, U1019 – UMR 9017 - CIIL, 59000 Lille, France; 5 Department of Rheumatology, MABlab ULR 4490, Univ. Lille, CHU Lille, 59000 Lille, France

**Keywords:** Osteoporosis, Fracture, Chronic obstructive pulmonary disease, Bone mineral density, Antiosteoporosis medications

## Abstract

**Summary:**

A retrospective study was conducted to calculate the cumulative incidence of hospital-initiated bone densitometry, in the year following hospitalization for fragility fracture in patients with chronic obstructive pulmonary disease. This cohort study demonstrated low rates of hospital-initiated bone densitometry with a 1-year cumulative incidence of 22.6%.

**Background:**

Osteoporosis is one of the most frequent comorbidities in chronic obstructive pulmonary disease (COPD) patients. A study was conducted to assess the management of osteoporosis in COPD patients using the INCLUDE health data warehouse.

**Objectives:**

The primary objective was to calculate the cumulative incidence of hospital-initiated bone densitometry, in the year following hospitalization for fragility fracture in COPD patients.

**Patients and methods:**

A retrospective, monocentric, observational study was conducted at Lille University Hospital with patients identified from January 2013 to December 2021. Patients with COPD, aged 40 or over, and hospitalized for a fragility fracture according to the ICD-10 classification were included. Bone densitometry was indexed according to French Common Procedures Classification (CCAM) acts by INCLUDE.

**Results:**

A total of 365 patients (~ 60% male, mean age 73.5 ± 12.3 years, and median Charlson score 2.0 (1.0; 4.0)) were included. Hospitalization units for fractures were orthopedics (*n* = 168), geriatrics (*n* = 46), rheumatology (*n* = 45), pneumology (*n* = 24), and others (*n* = 82). A total of 499 fractures were identified, most of them severe (hip (36.4%), vertebrae (30.1%), proximal humerus (11.5%), pelvis (10.7%), etc.). During the first year, 69 patients (18.9%) died, and 81 underwent hospital-initiated bone densitometry. The cumulative incidence of bone densitometry in the 1st year was 22.6% [CI 95% 18.3–27.1%]. Independent determinants of performing bone densitometry were female gender, low Charlson score, hospitalization in rheumatology, and vertebral fracture(s).

**Conclusion:**

The cumulative incidence of hospital-initiated bone densitometry, within 1 year of hospitalization for a fragility fracture in COPD patients was relatively low.

**Supplementary Information:**

The online version contains supplementary material available at 10.1007/s11657-025-01534-3.

## Introduction

Chronic obstructive pulmonary disease (COPD) is a debilitating condition that is associated with significant morbidity and mortality [1]. Osteoporosis is one of the most significant comorbidities of COPD and can lead to fragility fractures, including vertebral fractures (VCFs) [2–4]. This observation could be explained by the fact that patients with COPD have coexisting osteoporosis risk factors, such as the frequent use of inhaled or systemic corticosteroids, vitamin D deficiency, tobacco use, and chronic inflammation [5–8].

Osteoporosis in patients with COPD remains underdiagnosed and undertreated [9]. Currently, there are no official recommendations for the management of osteoporosis associated with COPD [10]. However, it appears necessary to screen for this comorbidity in patients with COPD, in order to identify patients most at risk of fracture, and therefore treat them with the standard drugs established for osteoporosis.

In this regard, a program called “BPCOS (*bronchopneumopathie chronique obstructive et os*),” based on the fracture liaison service (FLS) model, was implemented at Lille University Hospital in January 2017 to screen for osteoporosis in patients with COPD [11]. A FLS was already implemented at Lille University Hospital since January 2016 [12, 13]. In the BPCOS program, patients with COPD at any stage and a recent history of fragility fractures and/or severe COPD (group E with ≥ 2 exacerbations per year) are referred to a rheumatologist by a pneumologist, both working at Lille University Hospital. Furthermore, Lille University Hospital set up a health data warehouse in 2018 (INCLUDE), allowing the secure and anonymous collection of data produced during patient care. These data are used for several purposes, such as clinical research, improving the quality of care, and medico-economic analysis (https://include-project.chru-lille.fr/home-project/).

In this context, we conducted a retrospective study to evaluate osteoporosis care in patients with COPD at Lille University Hospital using the INCLUDE database. The main objective was to estimate the cumulative incidence of hospital-initiated bone densitometry performed in the year following hospitalization for a fragility fracture in a population of patients over 40 years of age with COPD. The secondary objective was to identify the determinants of hospital-initiated bone densitometry.

## Patients and methods

### Study design

A retrospective, single-center, observational study was conducted at Lille University Hospital, France, from January 1, 2013, to December 31, 2021. This research project was approved by the local medical research ethics committee.

### Study population

In this retrospective study, inclusion criteria were adults of both genders with COPD of any stage (group A, B, or E, according to GOLD ABE assessment tool), aged 40 years old or older, and hospitalized at Lille University Hospital for a fragility fracture, using the International Classification of Diseases 10th Revision (ICD-10) (Supplementary Table 1) [14]. Exclusion criteria were fractures associated with bone metastases and multiple index fractures (> 2 fractures) except for vertebral compressive fractures (VCFs). Out-patients with fragility fractures were also excluded.

### Study assessment data collection

All data were collected from the hospital data warehouse (INCLUDE) using French Common Procedures Classification (CCAM) and ICD-10 classification and completed by a full review of the computerized medical records of the patients.

### Patient demographics and comorbidities

INCLUDE warehouse allowed the identification of the age, gender, body mass index (BMI) ≥ 40 kg/m^2^, Charlson Comorbidity Index (CCI) (Supplementary Table 1), length of hospital stay and date of the index fragility fracture requiring hospitalization in the database.

Additional risk factors for osteoporosis and comorbidities were collected thanks to patients’ medical records, including current or past smoking, current or past alcohol abuse (defined as ≥ 3 units of alcohol per day for men, and ≥ 2 units for women), and major comorbidities such as diabetes mellitus, chronic kidney disease, and Parkinson’s disease. Because patients were not always hospitalized in medical departments, some risk factors were not collected (e.g., family history of fragility fractures, history of previous fragility factures, history of falls, age of menopause for women, use of medications associated with osteoporosis such as corticosteroids).

### Fragility fracture

The “Index fracture” was defined as the first fragility fracture occurring during the study period. A fragility fracture was defined as a low-trauma fracture, typically resulting from a fall from standing height or less. The types of fragility fractures included were vertebral compression fractures (VCFs), hip fractures, non-hip non-vertebral severe fractures (proximal humerus, pelvis, distal femur, proximal tibia, and at least three rib fractures), and non-severe fractures (e.g., wrist, elbow, and ankle fractures) [16]. Fractures were confirmed through radiographic readings at the time of the hospitalization for the index fracture. The circumstances of each fragility fracture (e.g., following a fall or not) and the hospitalization unit were recorded.

### Anti-osteoporotic medication

The available data on antiosteoporosis medications (AOM) were included, specifically whether patients received AOM prior to the index fracture and, if so, the type of AOM (zoledronic acid, oral bisphosphonates, teriparatide, or denosumab). Information on patients who started AOM following the index fracture was also collected.

### Biochemical parameters and bone densitometry

Biochemical parameters, including serum calcium, phosphate, parathormone, and 25-hydroxyvitamin (OH) D levels, were collected when available. The date of hospital-initiated bone densitometry was recorded.

Bone densitometry was coded using the French Common Procedures Classification (CCAM) (Supplementary Table 1). The department conducting the bone densitometry was documented.

### Definition of densitometric osteoporosis

Densitometric osteoporosis was diagnosed using the International Society for Clinical Densitometry (ISCD) official positions and the World Health Organization (WHO) classification [15]. For postmenopausal women and men aged 50 years or older, osteoporosis was defined as a *T*-score of ≤  − 2.5 SD at the lumbar spine, total hip, or femoral neck. Osteopenia was defined as a *T*-score between − 1.0 and − 2.5 SD. As there were only 11 patients between 40 and 50 years old, we decided to use the same definition based on *T*-scores.

### Pneumology department follow-up

For each patient, we determined whether they had prior follow-up in the Department of Pneumology. This included at least one hospitalization, external consultation, or respiratory functional exploration in this department before the index fracture.

### Statistical analysis

Categorical variables are expressed as frequency and percentage, and quantitative variables as means ± standard deviation in case of normal distribution or medians (interquartile range, IQR) otherwise. Normality of distributions was checked graphically and using the Shapiro–Wilk test.

The cumulative incidence of bone densitometry performed in 12 months was estimated using the Kalbfleisch and Prentice method considering death as a competing event. Potential risk factors for bone densitometry were assessed using a Fine and Gray competing risk regression model. The significant factors at the level of 0.20 were introduced into a multivariable model. Sub-hazard ratios and their 95% confidence intervals were derived from models.

Statistical testing was conducted at the two-tailed α-level of 0.05. Data were analyzed using the SAS software version 9.4 (SAS Institute, Cary, NC).

## Results

### Demographic data and baseline characteristics

A total of 471 patients identified by INCLUDE were eligible for inclusion in this study, of which 106 were excluded due to lack of fractures (*n* = 80), high-trauma fractures (*n* = 22), and rib fractures related to cardiac massage (*n* = 4). A total of 365 patients met the inclusion criteria (Supplementary Fig. 1).

**Fig. 1:**
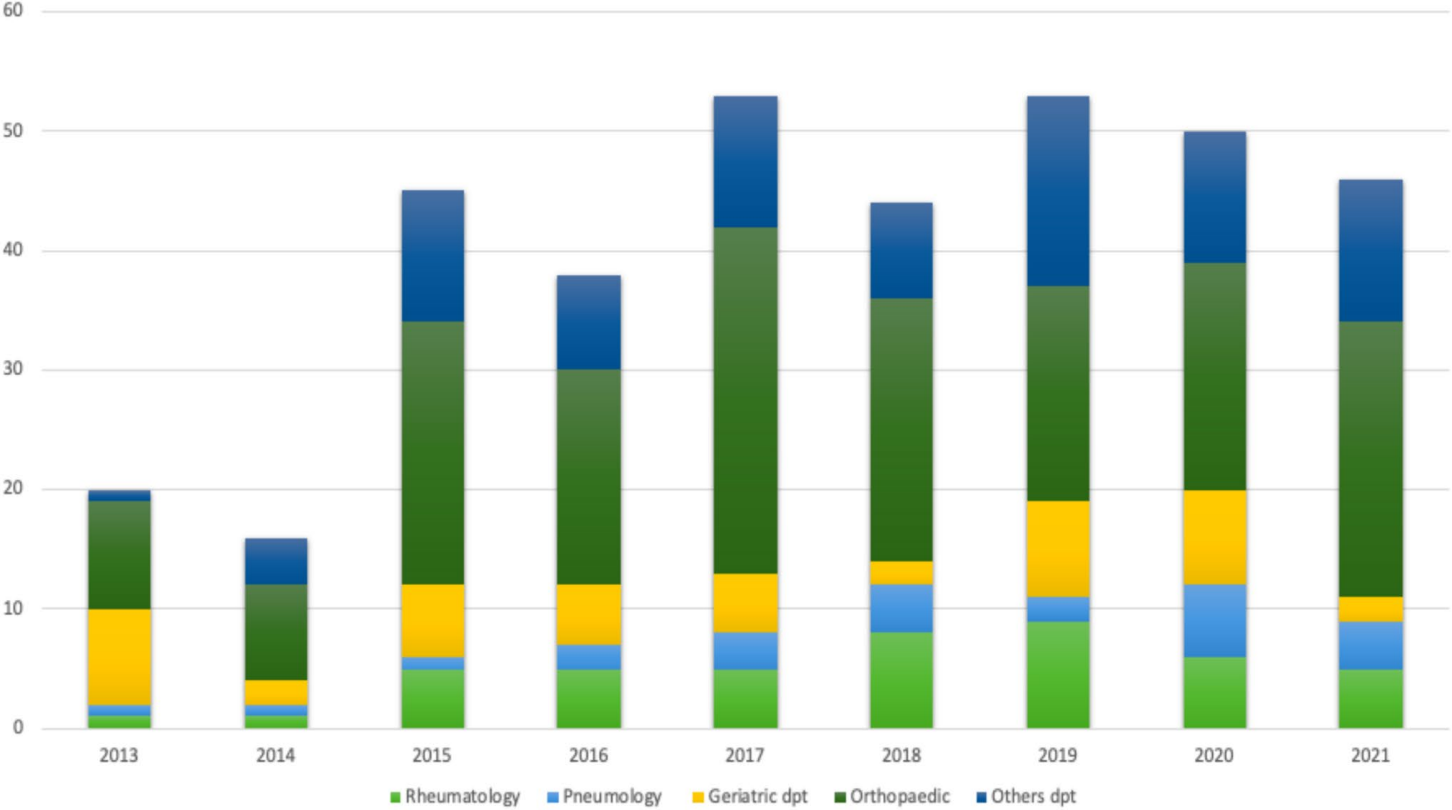
Distribution of hospitalized patients by year and unit of stay

Of the 365 patients (59.7% male, mean (SD) age 73.5 (12.3) years), 168 (46%) came from the orthopedic department, 46 (13%) from the geriatric department, 45 (12%) from the rheumatology department, 24 (7%) from the pneumology department, and 82 (22%) from other departments (e.g., thoracic surgery department, internal medicine department, and neurosurgery department) (Fig. 1). The mean (SD) length of stay at hospital was 14.6 (13.6) days. Almost half of the patients (*n* = 169/365, 46.3%) were already followed by the Pneumology department at Lille University Hospital before the index fracture.

### Patient’s comorbidities

Regarding comorbidities, 85 patients (23.3%) presented a history of diabetes mellitus, 72 (19.7%) with a history of heart failure, 60 (16.4%) with prior lower extremity peripheral artery disease, and 54 (14.8%) with a history of chronic kidney disease (Table 1). Median CCI score was 2.0 (1.0; 4.0) (Table 1).

**Table 1:** Baseline characteristics

Characteristics	*N* = 365
Male gender, *n* (%)	218 (59.7)
Age (years), mean ± SD	73.5 ± 12.3
Osteoporosis risk factors	
Active or weaned smoking, *n* (%)	268 (73.4)
Excessive alcohol consumption, *n* (%)	147 (40.3)
Comorbidities	
Charlson’s index, median (*Q*1–*Q*3)	2.0 (1.0; 4.0)
Diabetes mellitus, *n* (%)	85 (23.3)
Heart failure, *n* (%)	72 (19.7)
Lower extremity peripheral artery disease, *n* (%)	60 (16.4)
Chronic kidney disease, *n* (%)	54 (14.8)
Cerebrovascular accident, *n* (%)	36 (9.9)
Non-metastatic cancer, *n* (%)	35 (9.6)
Neurocognitive disorder, *n* (%)	34 (9.3)
Hepatopathy, *n* (%)	30 (8.2)
Hemiplegia, *n* (%)	24 (6.6)
Heart attack, *n* (%)	15 (4.1)
Rheumatic disease, *n* (%)	13 (3.6)
Metastatic cancer, *n* (%)	9 (2.5)
Severe obesity (BMI ≥ 40 kg/m^2^), *n* (%)	6 (1.6)
Parkinson’s disease, *n* (%)	5 (1.4)
Gastro duodenal ulceration, *n* (%)	2 (0.5)

### Fractures

Some patients had several fractures, and 499 fractures were found in 365 patients (Table 2). A fracture was related to a fall in 281 patients (77.0%). A total of 110 patients (30.1%) presented with at least one vertebral compression fracture (VCF), 133 patients (36.4%) had hip fractures, 42 (11.5%) had proximal humerus fractures, 39 (10.7%) had pelvis fractures, and 6 (1.6%) had distal forearm or wrist fractures.

**Table 2:** Characteristics and types of fracture

Characteristics of the fracture	Number
Severe hip fracture, *n* (%)	133 (36.4)
Severe vertebral fracture, *n* (%)	110 (30.1)
Thoracic vertebral fracture, *n* (%)	54 (14.8)
Lumbar vertebral fracture, *n* (%)	74 (20.2)
Other severe fracture, *n* (%)	110 (30.1)
Pelvis fracture, *n* (%)	39 (10.7)
Rib fracture (3 or more), *n* (%)	30 (8.2)
Fractures of the upper end of the humerus, *n* (%)	42 (11.5)
Fracture lower end of the femur, *n* (%)	1 (0.3)
Non-severe fracture, *n* (%)	39 (10.7)
Elbow, *n* (%)	5 (1.4)
Ankle, *n* (%)	1 (0.3)
Collarbone fracture, *n* (%)	3 (0.8)
Rib fracture (less than 3), *n* (%)	26 (7.1)
Wrist, *n* (%)	6 (1.6)
Tibia, *n* (%)	1 (0.3)
Fracture following a fall, *n* (%)	281 (77)

Of the 110 patients with at least one VCF, there was a total of 217 VCFs, for an average of 2.1 (± 1.6) fractures per patient.

Regarding the distribution of patients, 86.4% of patients with hip fractures were hospitalized in the orthopaedic department, while 37.3% of patients with a VCF were hospitalized in the rheumatology department and 24.5% in the geriatric department.

### Mortality

Of the 365 patients, 69 (18.9%) died during the first year of follow-up after the index fracture including 28 deaths (7.7%) during the initial hospitalization.

### Biochemical parameters and bone densitometry

Biochemical parameters (serum calcium, phosphate, parathormone, and 25OH vitamin D) were not available for all patients (Supplementary Table 2).

Of the 365 patients, 81 had hospital-initiated bone densitometry performed during the first year of follow-up (22.6%). Fifty-six were identified by INCLUDE while 25 patients with a bone densitometry were identified by the full reading of the computerized medical file of patients. Almost half of them were performed by the rheumatology department (*n* = 40, 49.4%) whereas 33 (40.7%) by the nuclear medicine department and 8 (9.9%) outside the Lille University Hospital (Table 3).

**Table 3:** Bone densitometry results

Bone densitometry results	*N* = 83	*N* (%)
Total hip	*N = 56*	
Normal Osteopenia Osteoporosis		16 (28.6) 24 (42.8) 16 (28.6)
Femoral neck	*N = 73*	
Normal Osteopenia Osteoporosis		8 (10.9) 33 (45.2) 32 (43.8)
Lumbar spine	*N = 80*	
Normal Osteopenia Osteoporosis		32 (40) 27 (33.7) 21 (26.2)

**Fig. 2:**
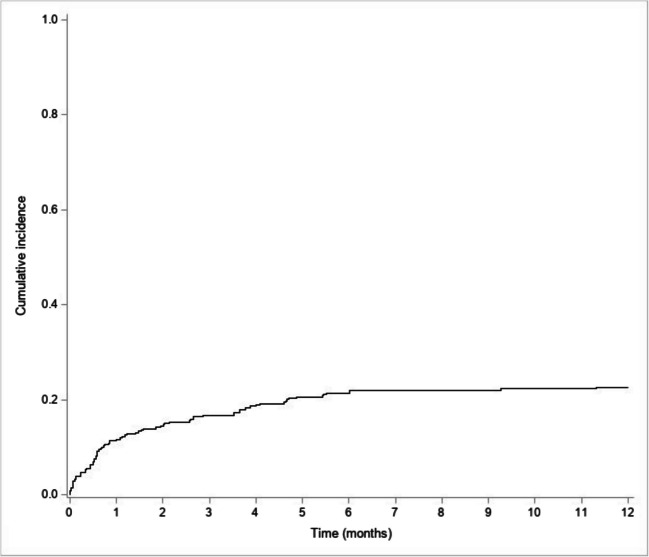
Cumulative incidence of bone densitometry

This study shows a 1-year cumulative incidence of hospital-initiated bone densitometry of 22.6% [95% CI 18.3–27.1%] (Fig. 2). Mostly all of them were performed within the first 6 months following the index fracture.

**Fig. 3:**
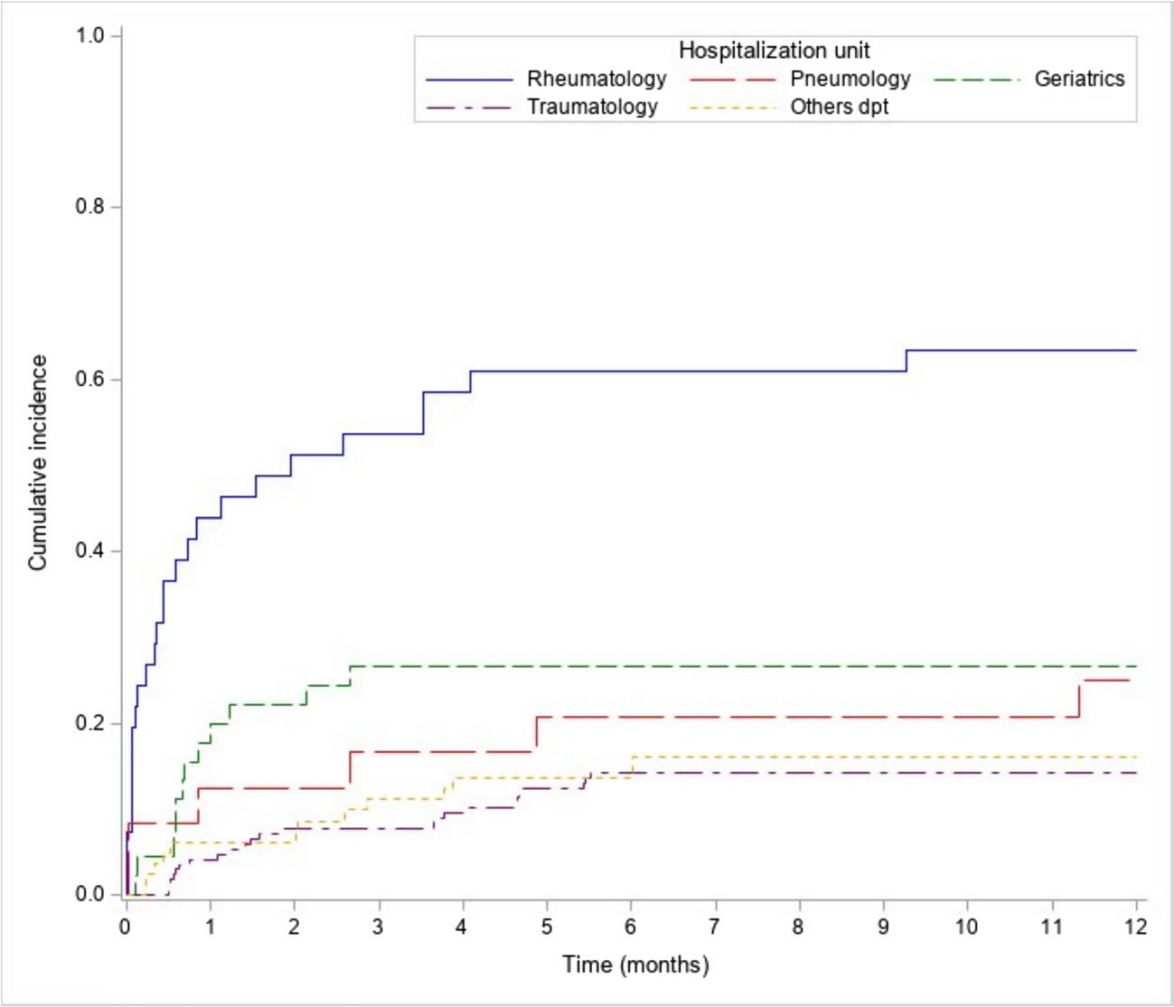
Cumulative incidence of bone densitometry by unit of stay

The 1-year cumulative incidence of bone densitometry was examined according to the hospitalization unit (Fig. 3) with a higher cumulative incidence found when patients came from the rheumatology department (63.4% [95% CI 46.3–76.4%]).

As reported in Table 4, bone densitometry performed at 1 year was independently associated with female sex, lower CCI, VCFs (vs. other fractures), and initial hospitalization in the rheumatology department (vs. other departments).

**Table 4:** Univariate and multivariate predictors of bone densitometry performed at 1 year in the 365 patients

	Hazard ratio	95% CI	*P*-value	Hazard ratio	95% CI	*P*-value
Age	1.18	(0.95; 1.45)	0.12	1.23	(0.94; 1.62)	0.13
Female	**1.59**	**(1.03; 2.46)**	**0.035**	**1.64**	**(1.01; 2.59)**	**0.044**
Tobacco consumption	1.00	(0.61; 1.63)	1.00			
Excessive alcohol consumption	0.96	(0.61; 1.51)	0.85			
Charlson Comorbidity Index	0.82	(0.64; 1.06)	0.12	**0.76**	**(0.58; 1.00)**	**0.047**
Hospitalisation unit			< 0.001			0.007
Rheumatology	Ref			Ref		
Pneumology	0.26	(0.10; 0.63)	**0.003**	0.65	(0.23; 1.78)	0.40
Geriatric dept	0.29	(0.14; 0.59)	**< 0.001**	0.31	(0.13; 0.70)	**0.004**
Orthopaedic	0.14	(0.07; 0.24)	**< 0.001**	0.51	(0.17; 1.45)	0.20
Other dept	0.16	(0.08; 0.31)	**< 0.001**	0.31	(0.14; 0.69)	**0.004**
Already under AOM	1.34	(0.54; 3.32)	0.53			
Hip fractures	**0.40**	**(0.23; 0.69)**	** < 0.001**	0.74	(0.27; 2.00)	0.55
Vertebral compression fractures	**5.03**	**(3.23; 7.82)**	** < 0.001**	**5.33**	**(1.93; 14.73)**	**0.001**
Non-hip non-vertebral fractures	**0.60**	**(0.35; 1.00)**	**0.048**	0.87	(0.41; 1.85)	0.72
Other fractures	0.92	(0.44; 1.90)	0.82			
Fracture related to a fall	0.66	(0.40; 1.07)	0.090	1.77	(0.99; 3.18)	0.054
Follow-up by pneumologists	0.92	(0.59; 1.42)	0.70			

Hazard ratios are expressed per 1 standard deviation increase for quantitative predictors

Abbreviations: *AOM* antiosteoporosis medications, *CI* confidence interval, *dept*: department

Bold values represent the best statistically significant results

The rheumatology department was the source of 38.4% of bone densitometry prescriptions, and 34.9% were prescribed by the geriatric department. The Lille FLS only allowed the prescription of 8.1% of bone densitometry and the BPCOS program 2.3%.

### Antiosteoporosis medications

Of the 365 patients, an AOM was prescribed for the first time in 92 (25.2%) patients in line with French guidelines (17,18). The main AOM prescribed were bisphosphonates (79.3%), followed by teriparatide (17.4%) and denosumab (3.3%). Seventeen patients (4.7%) were already on AOM at the time of the index fracture, and the AOM was changed in only five patients whereas the remaining patients (*n* = 12) stayed on the same treatment. Following the index fracture, 41 of 109 patients (37.6%) were under AOM without having performed hospital-initiated bone densitometry.

## Discussion

This cohort study demonstrated low rates of hospital-initiated bone densitometry among adults with COPD hospitalized for fragility fractures. The 1-year cumulative incidence was 22.6% [95% CI 18.3–27.1%], with a higher incidence observed following initial hospitalization in the rheumatology department (63.4% [95% CI 46.3–76.4%]). Factors independently associated with bone densitometry testing included female sex, lower CCI score, VCFs (vs. other fractures), and initial hospitalization in the rheumatology department (vs. other departments). Moreover, AOM was prescribed to 109 (29.9%) patients, including 41 patients without prior hospital-initiated bone densitometry testing.

The mean age of the patients was 73.5 ± 12.3 years, consistent with previous findings in our FLS cohort [12, 13]. The high proportion of men (59.7%) in this study could be explained by the selection of COPD comorbidity. Severe index fractures were predominant, with hip fractures (36.4%) and VCFs (30.1%) being the most common. While the prevalence of hip fractures in a COPD cohort may seem unexpected, it aligns with prior studies, including a large Veterans Health Affairs cohort where 47% of men undergoing hip fracture repair had COPD [19]. The high number of VCFs is consistent with previous evidence linking VCFs to COPD [20]. Mortality during the first year of follow-up reached nearly 20%, reflecting the advanced age, comorbidities, and severe fractures of this population. This aligns with prior findings on the increase in post-fracture mortality in COPD, particularly after hip fractures, due to a greater comorbidity burden [19].

Few studies in France have evaluated 1-year rates of bone densitometry and AOM initiation following fragility fractures in this population. In the FRACTOS study (356,895 patients aged ≥ 50 years hospitalized for severe fractures), only 5.3% of patients underwent bone densitometry testing and 16.7% received AOM [21]. Another nationwide study (177,000 patients aged ≥ 50 years hospitalized for severe and non-severe osteoporotic fractures), reported similarly low rates, with 10% undergoing bone densitometry and 15% receiving AOM [22, 23]. Our findings are consistent with the EPIFRACT study where 29.3% underwent BMD evaluated, and 10.5% received AOM [23], though these rates extended beyond the 1-year post-fracture time [24].

Regarding associated risk factors, female sex was significantly associated with bone densitometry testing, likely reflecting the underdiagnosis and undertreatment of osteoporosis in men [25, 26]. Two distinct profiles emerged: those hospitalized in rheumatology or geriatrics for VCFs and those hospitalized in orthopedics for hip fractures. Bone densitometry rates were significantly higher in the former group, likely due to greater involvement of rheumatology and geriatrics in osteoporosis care. Orthopedic departments had the lowest cumulative incidence of bone densitometry. This discrepancy underscores the different approaches to osteoporosis management between medical and surgical specialties. Rheumatology and geriatrics accounted for three-quarters of hospital-initiated bone densitometry testing, while contributions from the FLS and BPCOS program were limited. In addition, patients with higher CCI scores were less likely to undergo bone densitometry, possibly due to competing medical priorities and a lack of focus on osteoporosis in patients with multiple comorbidities [27].

To our knowledge, no other study has evaluated post-fracture hospital-initiated bone densitometry testing and AOM initiation in COPD patients. The lack of official guidelines for managing COPD-associated osteoporosis likely contributes to the underdiagnosis and undertreatment of this comorbidity [10].

### Study strengths and weaknesses

The strengths of this study include the following: (i) a large number of patients and the use of INCLUDE which allowed for data extraction using CCAM and ICD-10 codes; (ii) all medical records systematically assessed by a single investigator (EH) with experience in managing patients with osteoporosis; and (iii) this study being the first one, to our knowledge, to analyze hospital-initiated bone densitometry rates in COPD patients with fragility fractures.

Our study has several limitations. First, The hospital-based design limits generalizability to other populations, particularly outside tertiary care settings. Second, COPD characteristics and severity with spirometry results were not assessed, neither were medications (e.g., inhaled or systemic corticosteroids) and some osteoporotic risk factors (e.g., family history of hip fractures). Third, no biological evaluations (calcemia, phosphoremia, 25-hydroxyvitamin D, parathormone) were systematically performed in this study. Fourth, due to the retrospective design of this study, we did not systematically evaluate morphological vertebral fractures in all patients using conventional spine radiography or vertebral fracture assessment by DXA. Lastly, and this is probably the most important limitation, the rate of hospital-initiated bone densitometry using INCLUDE over a period of 1 year was assessed, but this does not include requests initiated in primary care. Finally, INCLUDE has its own limitations, as a high proportion of hospital-initiated bone densitometry testing was only found by the full reading of the patients’ computerized medical files.

## Conclusions

In this cohort study, the use of hospital-initiated bone densitometry testing following fragility fractures among patients with COPD remained low and was associated with female sex, Charlson index, type of fracture, and department of hospitalization. Given the deleterious impact of fractures on morbidity and mortality, strategies are needed to increase osteoporosis care in this population. Guidelines for bone health management in patients with COPD are required.

## Supplementary Information

Below is the link to the electronic supplementary material.

Supplementary file1 (DOCX 58 KB)

Supplementary file2 (DOCX 17 KB)

Supplementary file3 (DOCX 12 KB)

## Data Availability

The data that support the findings of this study are available from the corresponding author upon reasonable request.
