# Supplementary figures and images for: Association of hospital-initiated bone densitometry with hospitalization for fragility fracture at Lille University Hospital among adults with chronic obstructive pulmonary disease
Source: Arch Osteoporos. 2025 Apr 9;20(1):47. doi: 10.1007/s11657-025-01534-3 (PMC11982132; doi:10.1007/s11657-025-01534-3)

**Supplementary Figure 1: Study Flow Chart**


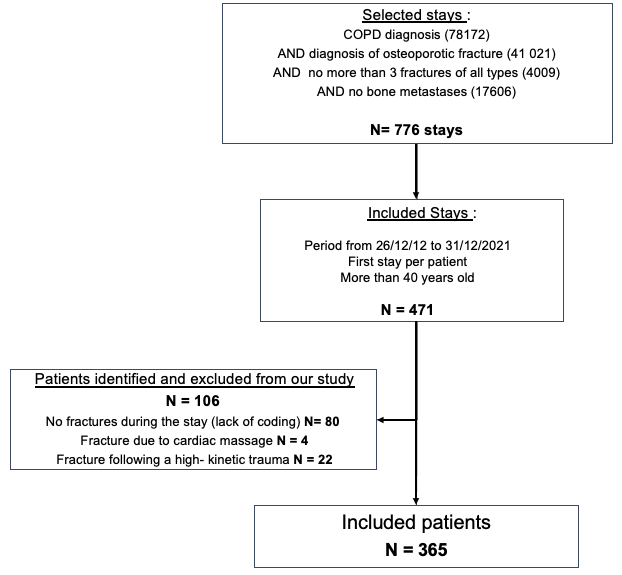

Supplement: Supplementary file 1 — Supplementary file1 (DOCX 58 KB) [file 11657_2025_1534_MOESM1_ESM.docx]
