# Supplementary material for: Association of hospital-initiated bone densitometry with hospitalization for fragility fracture at Lille University Hospital among adults with chronic obstructive pulmonary disease
Source: Arch Osteoporos. 2025 Apr 9;20(1):47. doi: 10.1007/s11657-025-01534-3 (PMC11982132; doi:10.1007/s11657-025-01534-3)
Supplement: Supplementary file 2 — Supplementary file2 (DOCX 17 KB) [file 11657_2025_1534_MOESM2_ESM.docx]

**Supplementary Table 1: The Classification of Diseases 10th Revision (ICD-10) and the French Common Procedures Classification (CCAM) used by INCLUDE**

|  | **ICD-10 or CCAM** |
| --- | --- |
| **Osteoporosis (unspecified, idiopathic, postmenopausal) with or without pathological fracture** | M800, M805, M8008, M8098, M815, M809, M819,  M8199, M8088, M8048, M8095 |
| **Chronic obstructive pulmonary disease** | J449, J448, J441 |
| **Hip fracture** | S720, S721, M844 |
| **Vertebral compression fracture** | S320, S3280, S2200, S3280, S3200, M485, M4850, M4856, M4956, M4846, M4857 |
| **Others fractures**  Pelvis fracture  Rib fracture  Fracture of the upper end of the humerus  Collarbone fracture  Wrist | S3280, S3230, S3240, S3250,  S2250, S2230, S2240  S4220  S4200  S5250 |
| **Bone densitometry** | PAQK007, PAQK900 |
| **Bone metastases** | C795 |
| **Severe obesity** | E668 |
| **Parkinson’s disease** | G20 |
| **Charlson Comorbidity Index** |  |
| **Myocardial infarction** | I21, I22, I252 |
| **Congestive Heart failure** | I099, I110, I130, I132, I255, I420, I425, I426, I427, I428, I429, I43, I50, P290 |
| **Peripheral vascular disease** | I70, I71, I731, I738, I739, I771, I790, I792, K551, K558, K559, Z958, Z959 |
| **Cerebrovascular disease** | G45, G46, H340, I6 |
| **Dementia** | F00, F01, F02, F03, F051, G30, G311 |
| **Chronic Pulmonary Disease** | I278, I279, J40, J41, J42, J43, J44, J45, J46, J47, J60, J61, J62, J63, J64, J65, J66, J67, J684, J701, J703 |
| **Rheumatic disease** | M05, M06, M315, M32, M33, M34, M351, M353, M360 |
| **Peptic ulcer disease** | K25, K26, K27, K28 |
| **Mild liver disease** | B18, K700, K701, k702, K703, K709, K713, K714, K715, K717, K73, K74, K760, K762, K763, K764, K768, K769, Z944 |
| **Diabetes without complication** | E100, E101, E106, E108, E109, E110, E111, E116, E118, E119, E120, E121, E126, E128, E129, E130, E131, E136, E138, E139, E140, E141, E146, E148, E149 |
| **Diabetes with complication** | E102, E103, E104, E105, E107, E112, E113, E114, E115, E117, E122, E123, E124, E125, E127, E132, E133, E134, E135, E137, E142, E143, E144, E145, E147 |
| **Hemiplegia or paraplegia** | G041, G114, G801, G802, G81, G82, G830, G831, G832, G833, G834, G839 |
| **Renal disease** | I120, I131, N032, N033, N034, N035, N036, N037, N052, N053, N054, N055, N056, N057, N18, N19, N250, Z490, Z491, Z492, Z940, Z992 |
| **Any malignancy** | C0, C1, C20, C21, C22, C23, C24, C25, C26, C30, C31, C32, C33, C34, C37, C38, C39, C40, C41, C43, C45, C46, C47, C48, C49, C50, C51, C52, C53, C54, C55, C56, C57, C58, C6, C70, C71, C72, C73, C74, C75, C76, C81, C82, C83, C84, C85, C88, C90, C91, C92, C93, C94, C95, C96, C97 |
| **Moderate or severe liver disease** | I850, I859, I864, I982, K704, K711, K721, K729, K765, K766, K767 |
| **Metastatic solid tumor** | C77, C78, C79, C80 |
| **AIDS HIV** | B20, B21, B22, B24 |
|  |  |
