# Supplementary material for: Association of hospital-initiated bone densitometry with hospitalization for fragility fracture at Lille University Hospital among adults with chronic obstructive pulmonary disease
Source: Arch Osteoporos. 2025 Apr 9;20(1):47. doi: 10.1007/s11657-025-01534-3 (PMC11982132; doi:10.1007/s11657-025-01534-3)
Supplement: Supplementary file 3 — Supplementary file3 (DOCX 12 KB) [file 11657_2025_1534_MOESM3_ESM.docx]

**Supplementary Table 2: Biochemical parameters**

| **Biochemical parameters** | **N** | **Mean ± SD** |
| --- | --- | --- |
| Calcemia (g/l) | N = 357 | 92.1 ± 5.8 |
| Phosphoremia (g/l) | N = 359 | 32.1 ± 7.8 |
| Parathormone (pg/ml) | N = 79 | 85.0 ± 12.3 |
| 25 OH vitamin D (ng/ml) | N = 142 | 23.6 ± 13.9 |
